# Knowledge Translation Task Force for core measures clinical practice guideline: a short report on the process and utilization

**DOI:** 10.1186/s43058-024-00580-1

**Published:** 2024-04-19

**Authors:** Marghuretta D. Bland, Jennifer L. Moore, Elizabeth Anderl, Megan Eikenberry, Arlene McCarthy, Geneviève N. Olivier, Tracy Rice, Amelia Siles, Hallie Zeleznik, Wendy Romney

**Affiliations:** 1 Physical Therapy, Neurology, & Occupational Therapy, Program in Physical Therapy, Washington University, St. Louis, MO USA; 2 Institute for Knowledge Translation, Carmel, IN USA; 3 Regional Center for Knowledge Translation in Rehabilitation, Sunnaas Rehabilitation Hospital, Oslo, Norway; 4 TIRR Memorial Hermann, Houston, TX USA; 5 College of Health Sciences, Physical Therapy Program, Midwestern University, Glendale, AZ USA; 6 PT, MS, DPT, Board Certified in Neurologic Physical Therapy, Former Program Director of Neurologic Physical Therapy Residency, Rehabilitation Services, Kaiser Permanente, San Francisco, CA USA; 7 Department of Physical Therapy and Athletic Training, University of Utah, Salt Lake City, UT USA; 8 Department of Human Performance, Division of Physical Therapy, West Virginia University, Morgantown, WV USA; 9 School of Health and Rehabilitation Services, Physical Therapy Division, The Ohio State University, Columbus, OH USA; 10 Strategic Initiatives and Professional Development, UPMC Centers for Rehab Services Pittsburgh, Pittsburgh, PA USA; 11 Department of Physical Therapy and Human Movement Science, Sacred Heart University, Fairfield, CT USA

**Keywords:** Physical therapy, Implementation, Clinical practice guideline

## Abstract

**Background:**

As part of the 2018 Clinical Practice Guideline (CPG): A Core Set of Outcome Measures for Adults with Neurologic Conditions Undergoing Rehabilitation, a Knowledge Translation (KT) Task Force was convened. The purpose of this short report was to (1) demonstrate the potential impact of a CPG KT Task Force through a practical example of efforts to implement a CPG into neurologic physical therapy practice and (2) describe the process to convene a KT Task Force and develop products (KT Toolkit) to facilitate implementation of the CPG.

**Methods:**

To describe the process used by the KT Task Force to develop and review a KT Toolkit for implementation of the CPG.

**Results:**

Utilizing the Knowledge-To-Action Cycle framework, eight tools were developed as part of the KT Toolkit and are available with open access to the public. Findings indicate that the Core Outcome Measures Homepage, which houses the KT Toolkit, has had greater than 70,000 views since its publication.

**Conclusions:**

This short report serves as an example of the efforts made to implement a CPG into physical therapy practice. The processes to facilitate KT and the tools developed can inform future implementation efforts and underscore the importance of having a KT Task Force to implement a CPG. Moving forward, KT Task Forces should be convened to implement new or revised guidelines.

**Trial registration:**

N/A.

**Supplementary Information:**

The online version contains supplementary material available at 10.1186/s43058-024-00580-1.

Contributions to the literature

- Pre-publication, a Knowledge Translation (KT) Task Force convened to support the implementation of a clinical practice guideline (CPG) that recommended a core set of outcome measures for adults undergoing neurologic rehabilitation.
- This manuscript outlines the process used by a KT Task Force to build a KT Toolkit and to assess the utilization of the toolkit.
- The processes to facilitate KT and the tools developed by this task force can inform future implementation efforts and underscore the importance of having a KT Task Force to implement a CPG.

## Background

Clinical practice guidelines (CPGs) contain evidence-based recommendations designed to assist practitioners and patients with health care decisions and achieve more uniform care delivery [1]. Research on the use of guideline recommendations in clinical practice highlights implementation challenges [2, 3] that should be considered. In a systematic review of the utilization of guidelines, the authors identified that clinicians did not use guideline recommendations about two-thirds of the time [4], while studies investigating methods to facilitate the adoption of guidelines have also highlighted many challenges to successful implementation, with some reporting minimal to no changes in practice after active implementation efforts [5–7].

Specifically in neurologic physical therapy, adoption of CPG recommendations in routine clinical practice is limited [8–14]. As CPG development and implementation efforts continue to grow, it is critical to understand the facilitators and barriers to using these guidelines, as well as the successes and shortcomings that occur during their implementation to maximize efforts.

The purpose of this short report was to (1) demonstrate the potential impact of a CPG Knowledge Translation (KT) Task Force through a practical example of efforts to implement a CPG into neurologic physical therapy practice and (2) describe the process to convene a KT Task Force and develop products (KT Toolkit) to facilitate implementation of the CPG.

## Methods

**Fig. 1:**
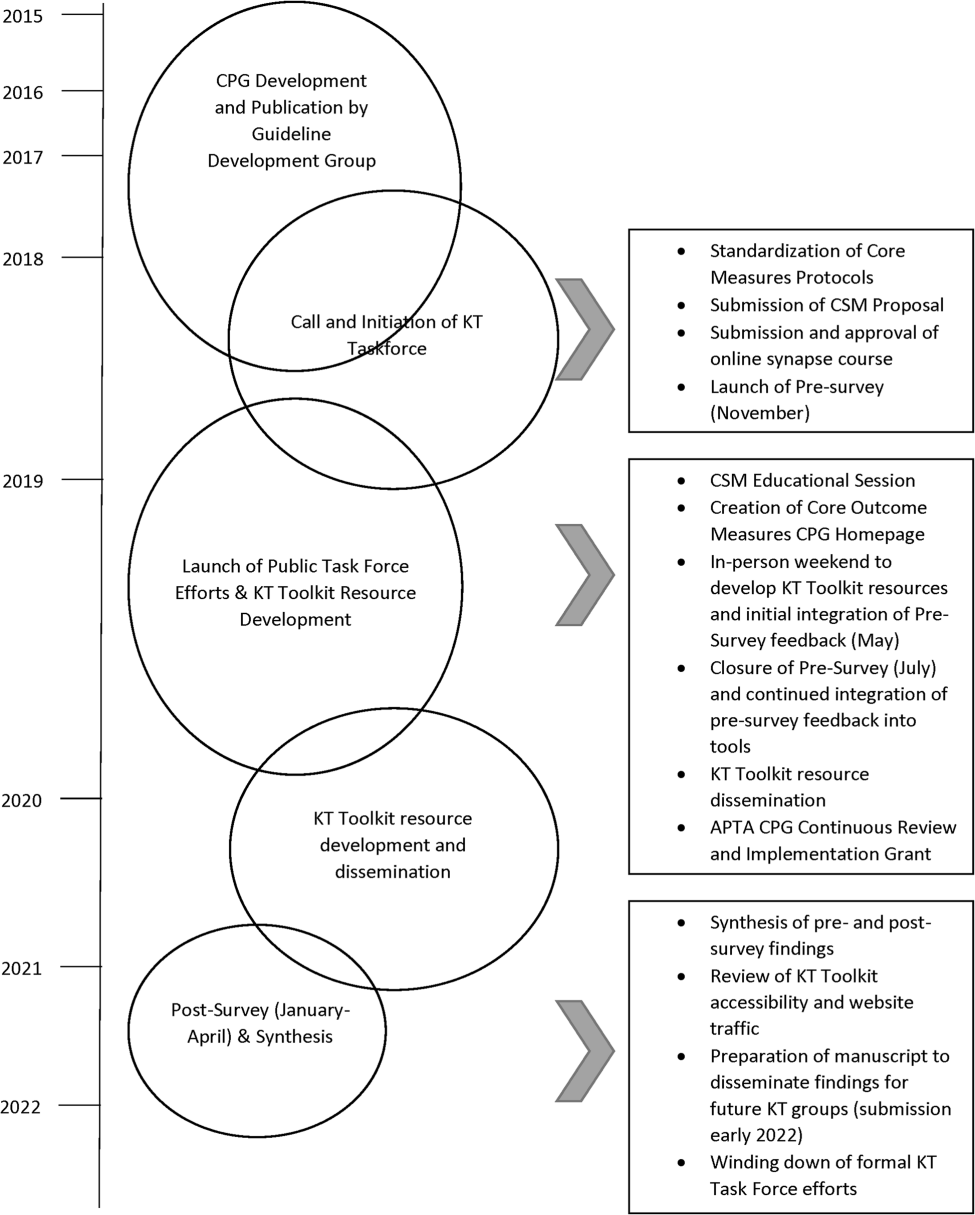
Timeline of Task Force efforts highlighting key KT Toolkit development steps as well as funding and synthesis of results. Size of circles approximates level of effort of Task Force members and circle overlap emphasizes the interconnection of Task Force efforts. CPG, clinical practice guideline; KT, Knowledge Translation; CSM, Combined Sections Meeting

With the planned publication of the 2018 Clinical Practice Guideline: A Core Set of Outcome Measures for Adults with Neurologic Conditions Undergoing Rehabilitation (Core Set) [9], the Academy of Neurologic Physical Therapy (ANPT) initiated a task force during the final steps of CPG development to assist with the dissemination and implementation of the Core Set. The KT Task Force for the Core Outcome Measures CPG (Task Force) commenced in 2017. In alignment with the ANPT Strategic Plan, the Task Force supported two ANPT strategic areas: (1) translating evidence into practice to ensure high-value care with input from practitioners to inform future research and (2) improving communications within ANPT and with providers of physical therapy to ensure access to education, networking, and key resources [15]. The Task Force includes a diverse volunteer group of expert physical therapists in background, geography, experience, and practice, and all members are board-certified specialists in neurologic physical therapy [16]. The timing of the Task Force allowed the team to work directly with the Core Set Guideline Development Group in the appraisal of the implementability of the Core Set. This step served as an effective way to learn the action statements as well as anticipate early implementation barriers of the Core Set. This can be seen visually by the overlap of circles at the top of Fig. 1. Figure 1 presents a visual of the workflow for the Task Force highlighting the timeline for the commencement of the group, development of the Knowledge Translation (KT) Toolkit, and synthesis and review of Task Force efforts.

Working in collaboration with the Core Set Guideline Development Group and the ANPT, the overarching goal of the Task Force was to support clinicians, educators, and organizations as each worked to implement the guidelines [9]. The Core Set recommends using six standardized outcome measures (Berg Balance Scale (BBS), Functional Gait Assessment (FGA), Activities-specific Balance Confidence Scale (ABC), 10-m walk test (10mWT), 6-min walk test (6MWT), and 5 times sit-to-stand (5TSTS)) to examine adults with neurologic disorders who have goals and the capacity to improve balance, gait, and transfers [9]. The specific goals of the Task Force were as follows: (1) to develop a KT Toolkit to assist with the implementation of the Core Set into neurologic physical therapy practice and education and (2) to assess utilization of the KT Toolkit in everyday neurologic physical therapy practice and education.

### KT Toolkit development

Once the Task Force convened, a KT expert who was a member of the Task Force (WR), educated the members on KT theory and provided KT guidance throughout the project. The Core Set called for KT interventions, also known as implementation strategies, to promote the adoption of guideline recommendations [9]. To accomplish this, the Task Force developed an implementation KT Toolkit, which is defined as a “packaged grouping of multiple knowledge tools and strategies that codify explicit knowledge” [17]. The Knowledge-to-Action Cycle (KTA), a knowledge translation framework, informed the development of the KT Toolkit [18]. This framework includes the development and publication of research and seven iterative phases (applicable phases and processes of the KTA framework italicized in the text below) to implement evidence into clinical practice [18]. Additionally, a key component of the framework is the identification of barriers, which guides the selection of KT interventions [18, 19].

The first KT Toolkit task was to establish standardized protocols for the administration of each core measure as each measure has various versions available [9]. The Task Force started with the *knowledge creation funnel* and *knowledge tools*of the KTA framework to create standardized protocols for each of the core measures [18]. Protocols were developed using the foundational resources in the Core Set, direct discussions with authors of the measures, expert opinion of the Task Force members, and by obtaining national-level feedback from stakeholders within and outside of neurologic physical therapy during a public comment period disseminated through the American Physical Therapy Association EDGE (Evidence Database to Guide Effectiveness) group and word of mouth. The final protocols were posted in an open-access format on the ANPT website allowing all stakeholders to view and download them [16]. The ANPT monitors the website’s user traffic, including the KT Toolkit and the Core Outcome Measures CPG homepage, with Google Analytics (http://www.google.com/analytics). The metrics include page views, unique page views, and exit rates (i.e., the user left the site immediately after viewing that page). Additionally, user statistics are monitored for each Synapse Education Center course which were created as part of the KT Toolkit.

After the standardized protocols were complete, the Task Force’s efforts shifted to developing additional tools to support the dissemination and implementation of the Core Set. Specifically, Task Force efforts focused on the CPG Action Statements 1–6 (Static and Dynamic Sitting and Standing Balance Assessment, Walking Balance Assessment, Balance Confidence Assessment, Walking Speed Assessment, Walking Distance Assessment, Transfer Assessment) and 8 (Use of the Core Set of Outcome Measures) of the CPG due to evidence level and recommendation strength [9]. The Task Force reviewed the KTA framework [18] again to determine strategies *to adapt the identified knowledge to local context*, *assess barriers to knowledge use*, *and to select, tailor, and implement interventions to promote the use of knowledge* [18]. Specific tools were prioritized to target previously published barriers (e.g., lack of knowledge and time) and facilitators (e.g., educational videos, resource sheet with information to interpret results) to outcome measurement use in allied health professions [20–25]. Additionally, before the final publication of the KT Toolkit resources, a Task Force member (WR) and Core Set Guideline Development Group member (JM) created a national stakeholder survey to formally examine the know-do gap [18] and assess barriers and facilitators to using the Core Set. Respondents provided closed and open-ended feedback on the type of tools clinicians and educators thought would be useful to facilitate the implementation of the Core Set. Survey results were compared to the KT Toolkit tools and resources in development, which affirmed the prioritization of the preliminary tools. These surveys comply with the Declaration of Helsinki standards as all respondents provided consent, and IRB approval was obtained through Sacred Heart University. Demographics of survey respondents can be found in Supplemental Table 1 and results of pre- and post-survey CPG and KT Toolkit utilization can be found in Supplemental Table 2. An in-depth discussion of survey results is beyond the scope of this short report.

## Results

### KT Toolkit

Table 1 specifically outlines each resource available in the KT Toolkit, including the goal of the tool, the *representative phase of the KTA* [18], and a description of how the tool may be utilized or implemented into a clinical or educational setting. For example, the Pocket Cards for Interpretation of Core Measure Scores are short reference guides for each measure that can “fit in your pocket.” They provide a brief overview of the standardized administration procedures and key values for interpreting the measure score. The barriers they address are (1) knowledge: decreased knowledge about how to administer the measure, (2) time: by having this short pocket reference the clinician can quickly reference the standardized protocol or the key values for interpretation of the measure, and (3) interpretation of scores: the pocket card provides value for both the clinician and patient in interpreting the measure score [22].

**Table 1:** Knowledge Translation (KT) Toolkit

**KT Tool**	**Goal of tool**	**Barrier**	**Knowledge-to-Action phase**^**18**^	**Potential uses—clinical**	**Potential uses—education**
**CSM 2019 Educational Session**	• Increase familiarity with and understanding of CPG and KT tools	Knowledge	• Adapt knowledge	• N/A	• N/A
**Recommended Standardized Administration of the Core Measures**	• Decrease variability in measure administration • Improve clinical utility • Provide additional guidance for administration in vague testing conditions	Knowledge	• Knowledge creation-knowledge tools	• Individual clinicians can utilize to improve personal administration and reduce variability • Practices can utilize to reduce variability across clinicians and sites	• Provide to students for reference when in clinical education • Provide students with strategies to become agents of change when participating in clinical education
**Quick Reference for Rehabilitation Professionals**	• Create a quick reference guide that provides key information for each action statement in the CPG	Knowledge	• Knowledge creation-knowledge tools	• Supplement to CPG education efforts • Post in common areas for reference	• N/A
**Quick Guide for Administration of all Measures**	• Provide a 1-page focused reference sheet with key information on the standardized administration protocols	Knowledge, time	• Knowledge creation-knowledge tools	• Access standardized protocols on one page and keep for quick reference (e.g.: clipboards, carts, desks, workstations) • Post in common areas for reference	• Provide to students for reference when in clinical education • Provide students with strategies to become agents of change when participating in clinical education and as future clinicians
**Pocket Cards for Interpretation of Core Measure Scores**	• Short reference guide for each measure that provides key values for interpreting the score of the measures and standardized administration	Knowledge, time, interpretation of scores	• Knowledge creation-knowledge tools	• Quick reference for interpreting and using scores obtained with core measures • Documentation anchored in published clinical values • Support clinical decision-making and recommendations • Post in common areas for reference	• Provide to students for reference when in clinical education • Provide students with strategies to become agents of change when participating in clinical education and as future clinicians
**Environmental Set Up for Core Measures**	• Addresses the barrier of measures taking increased time and space to complete • Provides a variety of examples across settings to set up measures • Includes one comprehensive equipment list for all core measures	Time, space	• Tailored interventions	• Set up clinical space and equipment to support the 6 core measures • Gather all necessary equipment for the measures into one area/space	• Set up lab spaces for practicing standardized administration of measures • Provide students with strategies to become agents of change when participating in clinical education and as future clinicians
**ANPT Synapse Center Online Courses**	• Create sustainable, open-access education that is active in nature and addresses the CPG action statements as well as the standardized protocols • Interactive courses that allow for self-assessment and standardization of core measures	Knowledge	• Adapt knowledge	• Utilize for clinical competencies • New hire training and education • Obtain CEUs	• Supplemental or asynchronous standardized education
**Knowledge Translation Report Card** **(a Simplified Version was published in Fall 2021)**	• Create a tool to support the action statements for shared decision-making • Increase the patient’s value of the outcome measures	Value, interpretation of scores	• Tailored interventions	• Utilize for patient engagement and shared decision-making • Patients can track change over time	• Educate students about patient engagement and shared decision-making • Educate students on key interpretation values for the measures • Educate students on communication with patients

Additionally, the Knowledge Translation Report Card for patient education was a tool that was added as a result of the pre-survey. Stakeholders identified “Resources for patients to understand the core set” and “Collaborative decision making with patients” as somewhat helpful to implement the core set into practice. Therefore, the Task Force developed the Knowledge Translation Report Card as a tool to facilitate shared decision-making among patients and clinicians.

### KT Toolkit accessibility and website traffic

The KT Toolkit is housed on the ANPT website, Core Set of Outcome Measures CPG homepage: https://www.neuropt.org/practice-resources/anpt-clinical-practice-guidelines/core-outcome-measures-cpg [16]. This open-access site allows the download of the materials free of charge. Additionally, the ANPT’s Education Center hosts the five free educational training courses designed to teach learners how to correctly administer each core measure: https://anpteducationcenter.org/ [26]. These interactive courses utilize multiple strategies such as self-assessment and video-based demonstrations to enhance learning.

Reviewing website use allowed us to *monitor knowledge use*. As shown in Table 2, the Core Outcome Measures CPG homepage is the top landing page (i.e., viewers go directly to that page) on the ANPT Website, behind the ANPT homepage for 2020 and 2021.

**Table 2:** ANPT website landing page views (viewers start directly on these pages)

**Date**	**ANPT homepage landing views**	**Core Outcome Measures CPG homepage landing views**	**Core Outcome Measures CPG homepage webpage ranking across ANPT site**
1/1/2021–11/30/2021	71,967	47,246	Second
2020	59,796	36,148	Second
2019	51,142	12,596	Eighth

The ANPT’s Synapse Education Center website which houses the Online Courses in the KT Toolkit, reports that the *Core Outcome Measures: 5TSTS and ABC* course has the highest number of learners (single-users), 1,072. The *Score Interpretation and Continuum Use* course is next with 837 learners. This is followed by the *BBS*, *10mWT*, and *6MWT*, and *FGA* courses with 655, 609, and 478 learners, respectively.

## Discussion

The outcomes of this KT Task Force’s efforts demonstrate how a group could facilitate the implementation of CPG recommendations. Specifically, this short report illustrates the importance of convening a KT Task Force prior to CPG release to support the implementation of a CPG and outlines the process of and materials produced for a KT Toolkit to assist in disseminating and implementing the Core Set into neurologic physical therapy. Additionally, ANPT site metrics indicate that consumers are utilizing KT Toolkit resources.

This Task Force followed recommendations by our KT expert to use theory to develop and implement the KT Toolkit [17]. Similar to the medical literature, our KT Toolkit includes various educational strategies, paper/electronic documents as well as online educational courses, to change physical therapy knowledge [17, 27, 28]. Looking specifically at the electronic resources, both the number of page views from the Core Outcome Measures CPG homepage and the number of learners enrolled in the ANPT’s Education Center courses, it is clear that users are going to and utilizing these online resources, however there is room to grow and improve.

To better understand usability of the KT Toolkit we examined other KT tools used in rehabilitation. The Rehabilitation Measures Database (RMD) is a free, web-based Knowledge Translation tool designed to support clinicians seeking information on over 200 standardized measures across multiple disciplines [29]. In 2014, the RMD reported an average of 1852 hits per day and is tracking use across multiple countries [29]. Most recently, the RMD reported from December 2022 through December 2023 that there were 1,630,887 total users and 1,542,426 new users [30]. Although views of the Core Outcome Measures CPG homepage are much lower than the RMD it is important to note a few key differences. Compared to RMD, the Core Outcome Measures CPG homepage is only for the Core Set (six measures), focuses on measures primarily used by physical therapists and assistants, and includes some environmental and implementation resources such as the Knowledge Translation Report Card which are not available on RMD. Additionally, a specific limitation of the website is that it did not allow tracking of downloads of the tools. So while we can track the number of views a page had, we cannot speak to whether information was downloaded.

As described by the KTA framework, it is important to continue to *monitor knowledge use* and to *evaluate outcomes* [18]. As part of this process, the Core Outcome Measures CPG homepage and the ANPT Synapse Center Online Courses may benefit from additional usability testing and improvements [31], however, establishing an open-access resource for clinicians and educators to obtain materials appears to be an important step toward improving the dissemination and implementation of a CPG’s findings.

Finally, while toolkits may assist the adoption of a practice, research suggests that additional components, such as facilitation and audit and feedback, may enhance the toolkit’s impact [32, 33]. This in addition to greater collaboration with informaticists, knowledge brokers [34], and implementation scientists will be vital to success of future CPGs [35] and should be strongly considered in the development of future KT Task Forces.

## Conclusions

A KT Task Force to implement a new or revised CPG may facilitate the successful adoption of the guideline recommendations. This short report describes the Task Force’s processes to aid in the dissemination and implementation of the Core Set including the many Core Set resources now available through the KT Toolkit, and the utilization of the Core Set and tools through ANPT site access metrics for the associated webpages. Moving forward, KT Task Forces should be convened to facilitate the implementation of new or revised guidelines.

### Supplementary Information

**Additional file 1: Supplemental Table 1.** Pre- and post-survey respondent demographics of CPG and KT Toolkit utilization.

**Additional file 2: Supplemental Table 2.** Descriptive pre- and post-survey findings of CPG and KT Toolkit utilization.

## Data Availability

The datasets used and/or analyzed during the current study are available from the corresponding author on reasonable request.
