# Supplementary material for: Knowledge Translation Task Force for core measures clinical practice guideline: a short report on the process and utilization
Source: Implement Sci Commun. 2024 Apr 19;5:43. doi: 10.1186/s43058-024-00580-1 (PMC11027410; doi:10.1186/s43058-024-00580-1)
Supplement: Supplementary file 1 — Additional file 1: Supplemental Table 1. Pre- and post-survey respondent demographics of CPG and KT Toolkit utilization. [file 43058_2024_580_MOESM1_ESM.docx]

Supplemental Table 1: Pre- and post-survey respondent demographics of CPG and KT Toolkit utilization.

| **Primary Role** | **Pre-Survey (n=302)** | **Post-Survey (n=166)** |
| --- | --- | --- |
| Academician | 10% (29) | 8% (13) |
| Physical Therapist | 67% (201) | 64% (107) |
| Manager/Supervisor/Leadership (no patient care responsibilities) | 2% (5) | 1% (1) |
| Physical Therapist Assistant | 0% (0) | 0% (0) |
| Both Academic and PT/PTA | 15% (45) | 17% (28) |
| Both Manager and PT/PTA | 7% (20) | 8% (14) |
| Student | 1%(2) | 2% (3) |
| **Age** | **Pre-Survey (n=243)** | **Post-Survey (n=136)** |
| 20-30 | 17% (41) | 22% (30) |
| 31-40 | 37% (90) | 36% (49) |
| 41-50 | 25% (61) | 18% (25) |
| 51-60 | 16% (38) | 20% (27) |
| 61-70 | 5% (12) | 4% (5) |
| >70 | 0% (1) | 0% (0) |
| **ABPTS Certification** | **Pre-Survey (n=242)** | **Post-Survey (n=136)** |
| Neurologic | 57% (139) | 63% (85) |
| Geriatric | 3% (8) | 4% (6) |
| Other (including multiple specializations) | 4% (10) | 3% (4) |
| **ANPT Member** | **Pre-Survey (n=244)** | **Post-Survey (n=135)** |
| Yes | 87% (213) | 93% (125) |
| **Years Practicing** | **Pre-Survey (n=242)** | **Post-Survey (n=135)** |
| 0-4 | 19% (45) | 19% (25) |
| 5-9 | 23% (55) | 27% (37) |
| 10-14 | 10% (25) | 13% (18) |
| 15-10 | 13% (31) | 10% (13) |
| >20 | 36% (86) | 31% (42) |
| **Work Setting (Self-Identified Primary Role of Clinician)** | **Pre-Survey (n=162)** | **Post-Survey (n=89)** |
| Acute Care Hospital | 9% (15) | 10% (9) |
| Acute Rehabilitation | 29% (47) | 26% (23) |
| Long Term Acute Rehabilitation Hospital | 2% (4) | 1% (1) |
| Sub-Acute Rehabilitation | 4% (6) | 5% (4) |
| Long Term Care | 0% (0) | 1% (1) |
| Outpatient-Hospital Based | 42% (68) | 45% (40) |
| Outpatient-Private | 9% (14) | 11% (10) |
| Home Health Care | 2% (4) | 0% (0) |
| Day Rehabilitation Center (offers 3+ hours across disciplines to one patient per day) | 2% (4) | 1% (1) |
