# Supplementary material for: Knowledge Translation Task Force for core measures clinical practice guideline: a short report on the process and utilization
Source: Implement Sci Commun. 2024 Apr 19;5:43. doi: 10.1186/s43058-024-00580-1 (PMC11027410; doi:10.1186/s43058-024-00580-1)
Supplement: Supplementary file 2 — Additional file 2: Supplemental Table 2. Descriptive pre- and post-survey findings of CPG and KT Toolkit utilization. [file 43058_2024_580_MOESM2_ESM.docx]

Supplemental Table 2: Descriptive pre- and post-survey findings of CPG and KT Toolkit utilization.

| **Knowledge of Core Set** | **Pre-Survey**  **Yes (responses/n)** | **Post-Survey**  **Yes (responses/n)** |
| --- | --- | --- |
| I know which measures are recommended by the Core Set | 80% (252/314) | 94% (159/170) |
| I find the Core Set useful for my practice | 85% (267/313) | 93% (158/169) |
| Has the Core Set changed your behavior | 57% (178/313) | 76% (127/167) |
| **Knowledge and Confidence** | **Pre-Survey**  **Strongly Agree or Agree** | **Post-Survey**  **Strongly Agree or Agree** |
| **Berg Balance Scale**  I know how to administer  I know how to interpret  I feel confident in communicating the results of the measure to others | 98% (185/188)  99% (185/187)  98% (185/188) | 100% (97/97)  100% (97/97)  99% (96/97) |
| **Functional Gait Assessment**  I know how to administer  I know how to interpret  I feel confident in communicating the results of the measure to others | 89% (167/188)  87% (164/188)  87% (163/187) | 95% (92/97)  94% (91/97)  94% (91/97) |
| **Activities-Specific Balance Confidence Scale**  I know how to administer  I know how to interpret  I feel confident in communicating the results of the measure to others | 86% (161/188)  84% (158/188)  81% (153/188) | 95% (92/97)  93% (90/97)  89% (86/97) |
| **10 meter Walk Test**  I know how to administer  I know how to interpret  I feel confident in communicating the results of the measure to others | 97% (183/188)  96% (181/188)  94% (177/188) | 98% (95/97)  99% (96/97)  98% (95/97) |
| **6-Minute Walk Test**  I know how to administer  I know how to interpret  I feel confident in communicating the results of the measure to others | 96% (181/188)  91% (172/188)  90% (170/188) | 99% (96/97)  99% (96/97)  99% (96/97) |
| **5 Times Sit-To-Stand**  I know how to administer  I know how to interpret  I feel confident in communicating the results of the measure to others | 98% (184/188)  91% (172/188)  90% (169/188) | 100% (97/97)  98% (95/97)  98% (95/97) |
| **Reported Utilization** | **Pre-Survey**  **Strongly Agree or Agree** | **Post-Survey**  **Strongly Agree or Agree** |
| How often do you use the Berg Balance Scale to measure static and dynamic sitting and standing balance?  Initial Evaluation  Discharge  One Time in Between  Acute  Chronic Stable  Chronic Progressive | 59% (149/252)  57% (144/252)  48% (120/250)  68% (166/244)  60% (140/233)  56% (132/234) | 58% (81/139)  57% (79/138)  47% (64/137)  70% (95/135)  65% (80/124)  64% (83/130) |
| How often do you use the Functional Gait Assessment to measure walking balance?  Initial Evaluation  Discharge  One Time in Between  Acute  Chronic Stable  Chronic Progressive | 53% (134/252)  56% (141/251)  48% (121/250)  54% (130/242)  61% (144/235)  54% (126/233) | 62% (86/139)  62% (86/139)  56% (76/136)  57% (77/136)  73% (93/128)  65% (85/130) |
| †How often do you use the Activities-Specific Balance Confidence Scale to measure balance confidence?  Initial Evaluation  Discharge  One Time in Between | 31% (77/252)  29% (73/251)  22% (55/251) | 37% (51/139)  35% (48/139)  30% (41/138) |
| How often do you use the 10 meter Walk Test to measure walking speed?  Initial Evaluation  Discharge  One Time in Between  Acute  Chronic Stable  Chronic Progressive | 75% (190/252)  74% (185/251)  67% (168/251)  75% (184/244)  80% (187/235)  75% (175/234) | 80% (111/139)  77% (107/139)  73% (101/139)  83% (114/138)  80% (102/127)  77% (101/131) |
| How often do you use the 6-Minute Walk Test to assess walking distance?  Initial Evaluation  Discharge  One Time in Between  Acute  Chronic Stable  Chronic Progressive | 45% (114/251)  48% (121/250)  41% (103/250)  45% (111/244)  57% (133/235)  48% (113/235) | 58% (81/139)  58% (81/139)  55% (76/139)  62% (84/136)  65% (82/126)  60% (78/129) |
| How often do you use the 5 Times Sit-To-Stand to measure transfers?  Initial Evaluation  Discharge  One Time in Between  Acute  Chronic Stable  Chronic Progressive | 19% (47/252)  19% (47/251)  14% (35/251)  62% (151/245)  60% (143/237)  60% (140/235) | 28% (39/139)  27% (38/139)  24% (22/139)  75% (103/138)  75% (95/127)  74% (96/129) |
| How often do you use the Core Set of OMs to assess change over time?  Initial Evaluation  Discharge  One Time in Between  Acute  Chronic Stable  Chronic Progressive | 63% (160/252)  65% (164/251)  59% (148/250)  65% (159/244)  65% (150/232)  63% (146/232) | 73% (102/139)  75% (104/139)  66% (92/139)  76% (102/134)  77% (95/123)  77% (97/126) |
| How often do you discuss OM results with patients?  Initial Evaluation  Discharge  One Time in Between  Acute  Chronic Stable  Chronic Progressive | 89% (225/252)  91% (228/251)  84% (210/250)  92% (229/248)  92% (220/238)  91% (217/238) | 91% (127/139)  94% (131/139)  84% (115/137)  95% (130/137)  95% (120/126)  95% (123/129) |
| How often do you use collaborative/shared decision making with patients?  Initial Evaluation  Discharge  One Time in Between  Acute  Chronic Stable  Chronic Progressive | 88% (221/251)  88% (220/250)  88% (217/248)  90% (222/248)  90% (214/238)  90% (215/239) | 92% (128/139)  91% (127/139)  88% (123/139)  92% (126/137)  93% (117/126)  92% (119/129) |
| **General Questions** | **Pre-Survey**  **Strongly Agree or Agree** | **Post-Survey**  **Strongly Agree or Agree** |
| I think it’s important to use all measures in the Core Set | 40% (107/271) | 54% (81/149) |
| I always follow the standardized procedure when completing outcome measures | 73% (159/218) | 75% (92/123) |
| In general, using standardized outcome measures is too time consuming | 11% (23/215) | 8% (10/123) |
| I teach the students the core set (% yes) | 70% (19/27) | 92% (11/12) |
| **KT Toolkit** | **Post-Survey**  **Yes** | |
| Have you used any of the resources or tools developed by the KT Task Force | 82% (76/93) | |
| **KT Toolkit**  **Of the participants reporting using the resources or tools, how valuable do you find these tools in your practice?** | **Post-Survey**  **Extremely Helpful or Very Helpful** | |
| Recommended Standardized Administration of Core Measures | 74% (63/85) | |
| Quick Reference for Rehabilitation Professionals | 74% (54/73) | |
| Quick Guide for Administration of all Measures | 82% (68/83) | |
| Pocket Cards | 65% (41/63) | |
| Environmental Set-up | 71% (55/77) | |
| ANPT Synapse Center Online Course  Have you taken (% yes) | 59% (29/49) | |
| KT Report Card | 58% (39/67) | |

Core Set = Core Set of Outcome Measures CPG

†Use of the Activities-Specific Balance Confidence Scale across Acute, Chronic Stable and Chronic Progressive Conditions was not assessed
